# A study of concentration changes of Protoporphyrin IX and Coproporphyrin III in mixed samples mimicking conditions inside cancer cells for Photodynamic Therapy

**DOI:** 10.1371/journal.pone.0202349

**Published:** 2018-08-31

**Authors:** Rainer Landes, Alfredo Illanes, Daniela Goeppner, Harald Gollnick, Michael Friebe

**Affiliations:** 1 Institut für Medizintechnik, Otto-von-Guericke University, Magdeburg, Saxony-Anhalt, Germany; 2 Clinic for Dermatology and Allergology, Justus-Liebig-University, Gießen, Hesse, Germany; 3 University Hospital Magdeburg, Magdeburg, Saxony-Anhalt, Germany; Massachusetts General Hospital, UNITED STATES

## Abstract

Photodynamic Therapy (PDT) using Aminolevulinic acid (ALA) could be an effective and minimally invasively applicable way to treat many different types of tumors without radiation and large incisions by just applying a light pulse. However the PDT process is difficult to observe, control and optimize and the dynamical relationships between the variables involved in the process is complex and still hardly understood. One of the main variables affecting the outcome of the process is the determination of the interval of time between ALA inoculation and starting of light delivery. This interval, better known as drug-light interval, should ensure that enough Protoporphyrin IX (PPIX) is located in the vicinity of functional structures inside the cells for the greatest damage during the PDT procedure. One route to better estimate this time interval would be by predicting PPIX from the dynamical changes of its precursors. For that purpose, in this work a novel optical setup (OS) is proposed for differentiating fluorescence emitted by Coproporphyrin III (CPIII) and PPIX itself in samples composed of mixed solutions. The OS is tested using samples with different concentrations in mixed solutions of PPIX and the precursor CPIII as well as with a Polymethyl methacrylate test sample as additional reference. Results show that emitted fluorescence of the whole process can be measured independently for PPIX and its precursor, which can enable future developments on PPIX prediction from the dynamical changes of its precursor for subject-dependent drug-light interval assessment.

## Introduction

**Fig 1:**
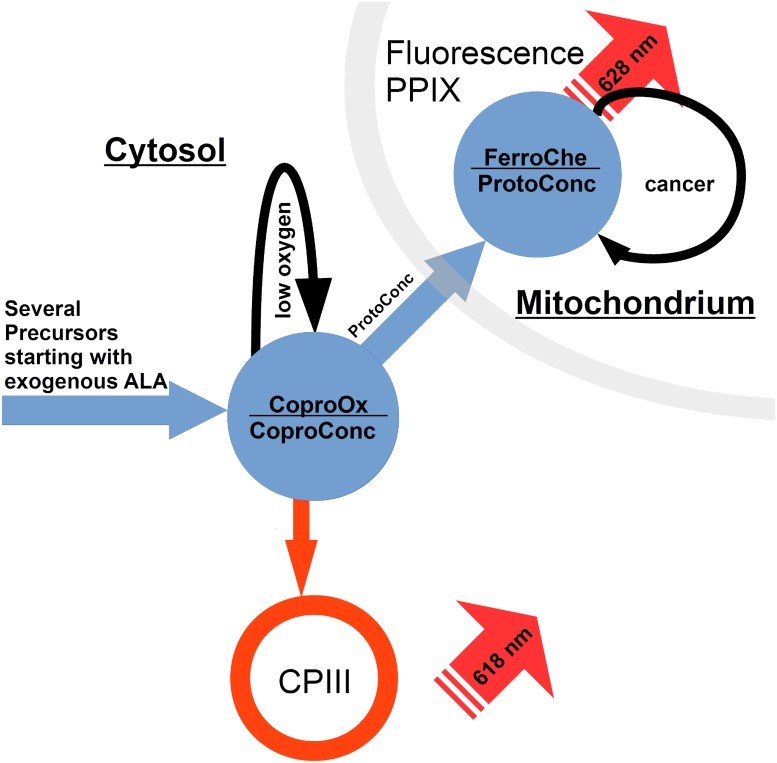
Scheme with the main steps of the heme synthesis for PPIX formation from ALA.

The main components of Photodynamic Therapy (PDT) are a photosensitizer (PS), oxygen and light with an appropriate wavelength. Since the 1980s studied extensively for different types of tumors. Up to 5.000 publications concerning PDT and about 250 officially reported clinical trials have been generated [1]. Although a positive response and good reproducibility was observed for nearly all types of solid tumors and PDT has been accepted as a treatment in various skin cancers, it is still in a niche within clinical practice for the treatment of cancers. The complex dynamical relationship between the involved variables makes the process difficult to monitor in its entirety which hinders optimization. The methods to apply PS can be categorized into two major groups: first endogenous PS that are generated by the cells themselves, stemming from a precursor molecule and show the highest selectivity, and exogenous PS that are applied to the targeted region or the system in the finished form and can only act from outside the cell. For endogenous PS the role of the metabolic pathway, the Heme Synthesis, is well established as it plays a major part in the accumulation of porphyrins in a number of cancer cells [2]. A PDT process starts with the transport of PS or Precursors inside the tumor, either by exogenously injecting the drug directly into the tumor or by systemic application either orally or via the bloodstream. The latter allows the optimal tumor selectivity of the endogenous route when using aminolevulinic acid (ALA), which is then gradually converted into photoactive molecules inside the tumor cells. In its natural form ALA already appears in the Heme-Synthesis yet not in concentrations as high as used in PDT. Coproporphyrin III (CPIII) which can accumulate outside the Mitochondria is one of the direct precursors of Protoporphyrin IX (PPIX) and can show fluorescence which is detectable. Its metabolic form, Coproporphyrinogen III is converted into Protoporphyrinogen III at the transfer into the Mitochondria which then is converted to PPIX and in the last step the transfer of an iron ion by the enzyme Ferrochelatase converts PPIX in Heme. Cancer cells show an accumulation of PPIX due to inactivation or downregulation of Ferrochelatase (Fig 1).

The PS targeted by the endogenous approach is PPIX. The endogenous approach with the application of ALA is the basic topic of this work. The ultimate goal of PDT is the generation of Reactive Oxygen Species (ROS), which are able to destroy the tumor cells. In the course of treatment the targeted cells can either die off due to apoptosis, necrosis or cause an inflammatory reaction by the immune system. If one of the main PDT components is not present, the reaction will not take place and no ROS will be generated. ROS have an extremely short lifetime, which means that the diffusion range is also extremely short (10-55nm) [3]. Thus, it is not only important to generate ROS, but also to generate them at the right location, namely in the vicinity of functional structures inside the mitochondria in order for them to cause the greatest damage. It is therefore crucial to know if enough PS is in the tumor and if it is located at the right location before the start of irradiation [3–6]. For this reason it is important to understand the transformation process of ALA to PPIX. If the treatment starts too early or too late, a sufficiently high concentration of PPIX, to produce ROS in strategic locations inside the cell, cannot be ensured. This interval of time between the ALA inoculation and the light delivery is known as “drug-light interval” and is one of the main factors influencing the outcome of a PDT procedure [1]. Nowadays the time for the drug-light interval generally used in clinical applications has been taken following the literature, where the interval has been adjusted for each patient equally [6]. Based on the fluorescence signal of PPIX, the chronological sequence of the intensity values of the fluorescence provides information about this drug-light interval by assuming the biggest peak as the optimal time for radiation. However, just fluorescence by itself gives no further information about the actual position of porphyrins inside the cells. Just the general presence of porphyrins is not enough information to guarantee a high success rate [3–5, 7]. Since the metabolic pathway of PPIX synthesis is subject to a number of factors that can vary from patient to patient, the drug-light interval should not be assumed to be the same for each patient. Simply waiting for an extended period of time and only looking for fluorescence as the starting point for treatment would give no clear information about the actual localization of PPIX inside the tissue. Waiting too long can mean that the majority of fluorescence stems from porphyrins that have already been excreted from the cells and accumulated in the intercellular volume, around the cells or in the intracellular volume, the cytosol. Targeting PPIX for treatment, at the exact time when it is produced inside the mitochondria is likely the best time to begin treatment since the mitochondria of cancer cells should be the main target to cause apoptosis. This can be deduced specifically by the amount of energy needed to cause the comparable cell death rates between endogenous and exogenous applications [3–7]. At the stage of extracellular porphyrin accumulation, the photosensitizer can destroy cancer cells but is far less effective for PDT than intracellular Porphyrin since the outcome is less predictable. We propose a new method to ensure this localization of PPIX by monitoring the fluorescence resulting by the direct chemical precursor of PPIX, CPIII, which is formed still outside the mitochondria. An increase in fluorescence signal that can be ascribed to this precursor, directly followed by fluorescence that can only come from PPIX, is likely to show the best starting point for the begin of therapy. For that a constant monitoring of the two precursors and of the PPIX is required and a specialized Optical Setup (OS) is needed for performing differentiation of PPIX and its precursor. So far separate detection methods for the different porphyrins rely on preparing tissue samples after biopsy, to detect different porphyrins. In extracts using High Performance Liquid Chromatography (HPLC) which can give hints about the amount of porphyrins to be expected but does not allow in vivo or in situ detection for online monitoring of concentrations [8]. Other approaches concentrate either on microscopic detection of buildup in cell solutions or are focusing solely on PPIX and not the precursors [9, 10]. In this work we present a novel OS enabling us to differentiate PPIX from its its direct precursor. It involves the monitoring of both involved porphyrins in mixed solution at the same pH, concentrating on the main emission peaks between 600 and 650 nm, after excitation at 405 nm. The main component of the OS is a dynamic high pass filter whose edge can be adjusted within a range of 617-704 nm. The OS has been tested using a sample with different concentrations of PPIX and CPIII as well as with a Polymethyl methacrylate (PMMA) test sample as a reference. The primary benefit of this approach will be the capability to detect PPIX and CPIII separately with CPIII being generated outside the mitochondria while PPIX is generated inside the mitochondria. This will allow us to detect and localize changes in concentration of porphyrins with the additional information about the position of localized concentration changes inside the cells. If the concentration of the precursors CPIII increases measurably and separately from other fluorescence sources, then a following increase in concentration of PPIX can only stem from new PPIX being produced inside the mitochondria. Further work will target the dynamic changes in concentration due to constant syntheses of porphyrins inside the cells in order to track the dynamical changes of precursors and PPIX.

## Materials and methods

In order to be able to predict PPIX levels both of its precursors, Uroporphyrin III (UPIII) and CPIII are of importance but since the main emission peaks of CPIII and Uroporphyrin III are only marginally different from one another, separated by less than 5 nm, and UPIII can be expected to only appear in smaller concentrations than CPIII, inclusion of UPIII has been omitted in this study [11, 12]. Our main objective with this work was to develop an optical device able to distinguish between PPIX and CPIII under conditions similar to the intracellular environment of cancer cells [13]. For this purpose a filter fluorometer, further on named Optical Setup (OS), was designed to measure fluorescence at different wavelengths. In order to determine the sensitivity of our OS, experiments were devised to test the ability to monitor and distinguish PPIX and CPIII at different concentrations in mixtures of both porphyrins. The validity of our experimental results was evaluated using a commercial spectrofluorometer (Cary Eclipse Fluorescence Spectrophotometer, Agilent, USA) as reference. In this section the OS, the preparation of the samples the measurement protocols and the control measurements with the commercial spectrofluorometer will be discussed and explained.

### Optical setup

**Fig 2:**
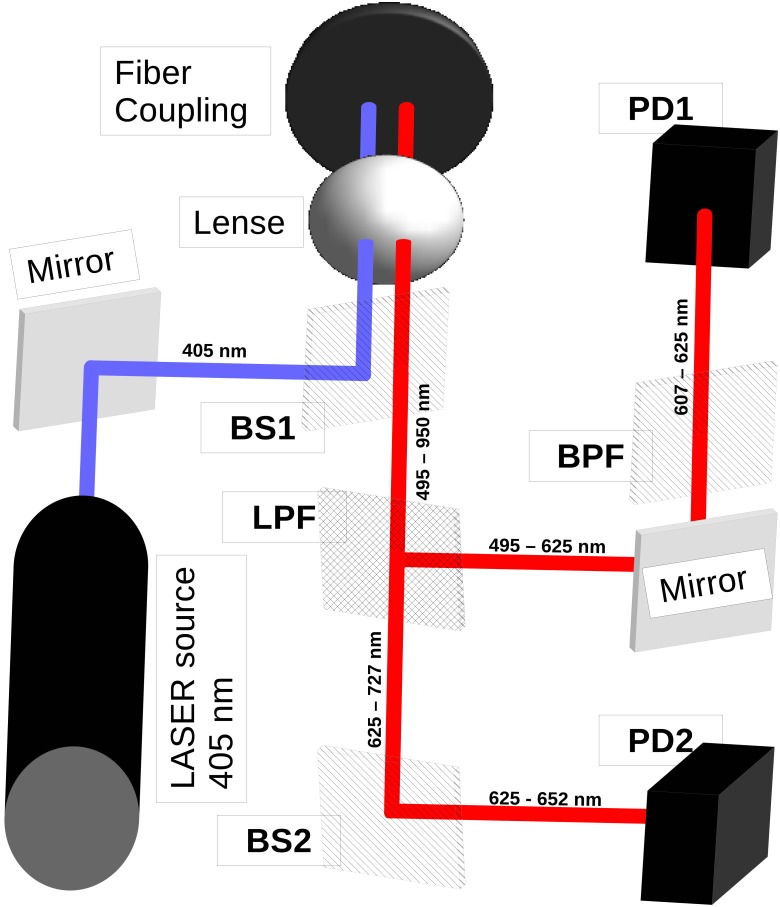
Schematic of the OS showing the light path after each filter and the change of the spectral range. blue: excitation beam, red: emission stemming from the excited samples, (description of abbreviations are shown in Table 1).

As a filter fluorometer the OS has two main functions: excitation and detection of the fluorescence of a mixture of porphyrins which are to be distinguished. A diagram of the OS with the beam path outwards (blue) and the beam path of the signals inwards (red) is shown in Fig 2.

**Table 1:** Main functional components of the OS.

Laser source	Laser	405 nm (excitation)	PPM110 405-125, Power Technology, USA
BS1	Beamsplitter 1	reflectance: < 495 nm transmittance: > 495 nm	FF495-Di03-25x36, Semrock, USA
LPF	longpass filter	at 57°: reflectance: < 625 nm transmittance: > 625 nm	TLP01-704-25x36, Semrock, USA
BPF	band-pass filter	Bandpass: transmittance: 607–645 nm	FF02-632/22, Semrock, USA
BS2	Beamsplitter 2	reflectance: < 652 nm transmittance: > 652 nm	FF650-Di01-25x36, Semrock, USA
PD1/PD2	Photodiode 1/2	15 mm^2^ active area, 12 ns respond time for wavelengths 430–900 nm	OSD15-5T, Centronic, United Kingdom

As source of excitation, a laser diode (PPM110 405-125, Power Technology, USA) with a central emission wavelength of 405 nm was used. The laser beam was channeled into an optical fiber (Laser Fiber GF300, World of Medicine, Germany) using a beamsplitter (BS1—FF495-Di03-25x36, Semrock, USA), which has a high reflectance for incident light with a wavelength below 495 nm and a high transmittance for light with a wavelength above 495 nm. The tip of the optical fiber in which the excitation beam is channeled was positioned inside a sample containing both CPIII and PPIX, which also allowed us to channel the fluorescence emitted by the samples and transmit it back to the OS. Since the beamsplitter BS1 has high transmittance at wavelengths higher than 495 nm, the light emitted by the sample is transmitted through this beamsplitter. To be able to separate the targeted spectral components of CPIII and PPIX contained in the emitted fluorescence, a long-pass filter (LPF—TLP01-704-25x36, Semrock, USA) was introduced into the signal beam path (Fig 2, red line), whose reflectance and transmittance were dependent on the light’s incident angle towards the filter surface. Transmittance and reflectance of this filter depend on the angle at which the incident light hits the surface of the filter. This tunability is of key importance to distinguish between PPIX and CPIII since the position of the main peaks of their emission can change depending on the conditions inside their environment. To decide the angle of this filter, small samples of the porphyrins in solution were prepared and measured with a spectrophotometer (Cary Eclipse Fluorescence Spectrophotometer). Following this procedure the position of the main peaks was determined to be 618 nm for CPIII and 628 nm for PPIX, hence the angle of the tunable filter (LPF) was set to 57° in order to reflect light with a wavelength lower than 652 nm and to have a high transmittance for light with longer wavelengths (> 625 nm). The exit beam from BS1 was split by the LPF into a reflected beam and a transmitted beam. The reflected beam contained spectral bands from 495–625 nm and 760–950 nm while the transmitted beam contained bands from 625–760 nm. Since both beams contained spectral components outside the range we are interested in, additional filters were introduced to narrow the contained spectral bands down. One additional filter (BPF- FF02-632/22, Semrock, USA) was set to filter the reflected beam and only leave the spectral range from 607–625 nm to reach Photodiode 1 (PD1, OSD15-5T, Centronic, United Kingdom). For the transmitted beam a second beamsplitter (BS2—FF650-Di01-25x36, Semrock, USA) was also used to filter out unwanted components so that only a spectral band from 625–652 nm could reach Photodiode 2 (PD2, OSD15-5T, Centronic, United Kingdom) (see Fig 2).

### Photodiode fluorescence data acquisition

The final filtered fluorescence was measured with the two PDs mentioned before and the data acquisition was achieved with a NI6009 (National Instrument, United States) card. A Labview interface was implemented for controlling the data acquisition main parameters and to display the obtained signals in real-time for both PDs. During the experiments the sampling frequency was adjusted to 1 Hz and the recording time was 30 seconds for each sample. With the CPIII main emission peak being located in the lower spectral band and the PPIX peak being located in the upper spectral band, a stronger change of intensity in the lower band would indicate a change in CPIII concentration, while a stronger change in the upper band would indicate a change in PPIX concentration.

### Chemicals for sample implementation

**Fig 3:**
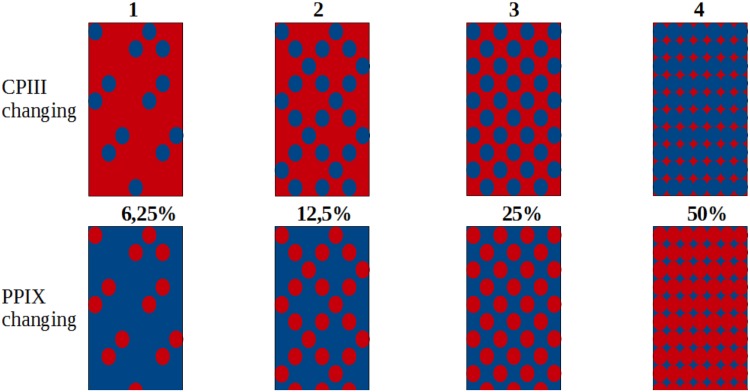
Protocol for the sample mixtures. For each type of sample one concentration was kept constant while the other was gradually doubled. Dots represent the changing constituent while the background colour represents the constant constituent.

We used two types of samples, type A where the concentration of CPIII (0.01g C654-3 Coproporphyrin III dihydrochloride, Inochem, United Kingdom) varied while the concentration of PPIX (258385-250MG Protoporphyrin IX, Disodium Salt, Sigma-Aldrich, Germany) was kept static and type B where the concentration of PPIX varied and the concentration of CPIII was kept static. The varying concentration always started with 15.47 *μ*Mol/l and was doubled until it reached 123.78 *μ*Mol/l as the highest concentration (Fig 3).

For both, A-type and B-type samples there were three testruns for each sample with different concentrations. This allowed us to repeat each measurement three times under comparable conditions. The main objective of these experiments was to prove the capability of our device to distinguish varying concentrations of the two porphyrins in relation to each other but most importantly separately from eachother. To prepare the varying concentrations in the samples, dilution series were created in four steps, starting with the lowest concentration at 15.47 *μ*Mol/l. For each consecutive step the concentration was doubled to get 123.78 *μ*Mol/l in the fourth sample. In the first three sets the PPIX concentration was kept static at the highest concentration of 123.78 *μ*Mol/l while the CPIII concentration changed from 15.47 *μ*Mol/l to 123.78 *μ*Mol/l as the highest concentration. In the second set the CPIII concentration was kept at 123.78 *μ*Mol/l while the PPIX concentration was gradually changed from 15.47 *μ*Mol/l to 123.78 *μ*Mol/l (see Fig 3). Two stem solutions were prepared with equivalent concentrations at 247.56 *μ*Mol/l at a pH of 7.4. To confirm the initial peak position of the used porphyrins, the individual stem solutions were analyzed in the Cary Eclipse fluorescence spectrophotometer. Following instructions from the suppliers, the PPIX salt had to be dissolved in a strong acid hence 3.5 mg were dissolved in a volume of 2 ml of one molar HCl. Following the dissolution of the salt the solution was titrated with one molar NaOH to neutral and the remaining volume was filled up to 20 ml with a 50% (v/v) mixture of PBS (VWRVE404-100TABS, VWR, Germany) and Methanol (8388.1, Carl Roth GmbH + Co. KG, Germany). The procedure for the CPIII salt was almost the same with only the first step reversed, where 2 ml of a one molar NaOH solution are used to dissolve the 10 mg CPIII dihydrochloride and one molar HCl was used to titrate to neutral pH. Following titration this solution had only a concentration of 247.56 *μ*Mol/l at 16.65 ml. To not further dillute it was decided to adjust the PPIX solution to this concentration by adding 3.31 ml of PBS—methanol mixture. In the end we could use 23.31 ml of PPIX stem solution and 16.65 ml of CPIII stem solution. In order to get the desired samples, the mixtures could now be prepared with concentrations according to the Tables 2 and 3.

**Table 2:** Sample preparation for varying CPIII concentrations [*μ*Mol/l].

	1	2	3	4
CPIII	15.47	30.95	61.89	123.78
PPIX	123.78	123.78	123.78	123.78

**Table 3:** Sample preparation for varying PPIX concentrations [*μ*Mol/l].

	1	2	3	4
PPIX	15.47	30.95	61.89	123.78
CPIII	123.78	123.78	123.78	123.78

For the static concentration 1 ml of stem solution was filled into an eppendorf test tube. For the changing concentrations the volumina were 0.125 ml for the lowest, 0.25 ml for the next highet, 0.5 ml for the second highest and finally 1 ml for the highest concentration. Each time, every sample had to be filled up to 2 ml except for the one with the highest concentrations. All in all these experiments used up 5.875 ml of PPIX stem solution and 5.875 ml of CPIII stem solution.

### Measurement protocol

**Fig 4:**
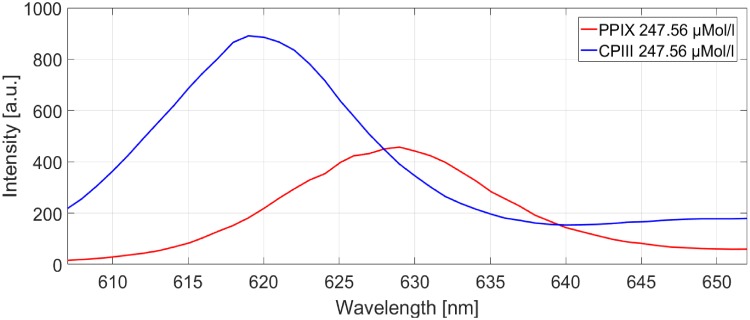
Position of the main peaks of the two porphyrin stem solutions. Measured at pH 7.4 and a concentration of 247.56 *μ*Mol/l.

The first measurements were conducted with the Cary Eclipse Fluorescence Spectrophotometer. For these measurements 1 ml of PPIX stem solution and 1 ml of CPIII stem solution were filled in crystal cuvettes and placed in the measurement chamber of the Spectrometer. The samples were excited with light of a wavelength of 405 nm and the spectrum was recorded between 500 and 700 nm with a step width of 5 nm. The position of the main emission peak of PPIX was located at 628 nm and the position of the main peak of CPIII was located at 617 nm (Fig 4).

**Fig 5:**
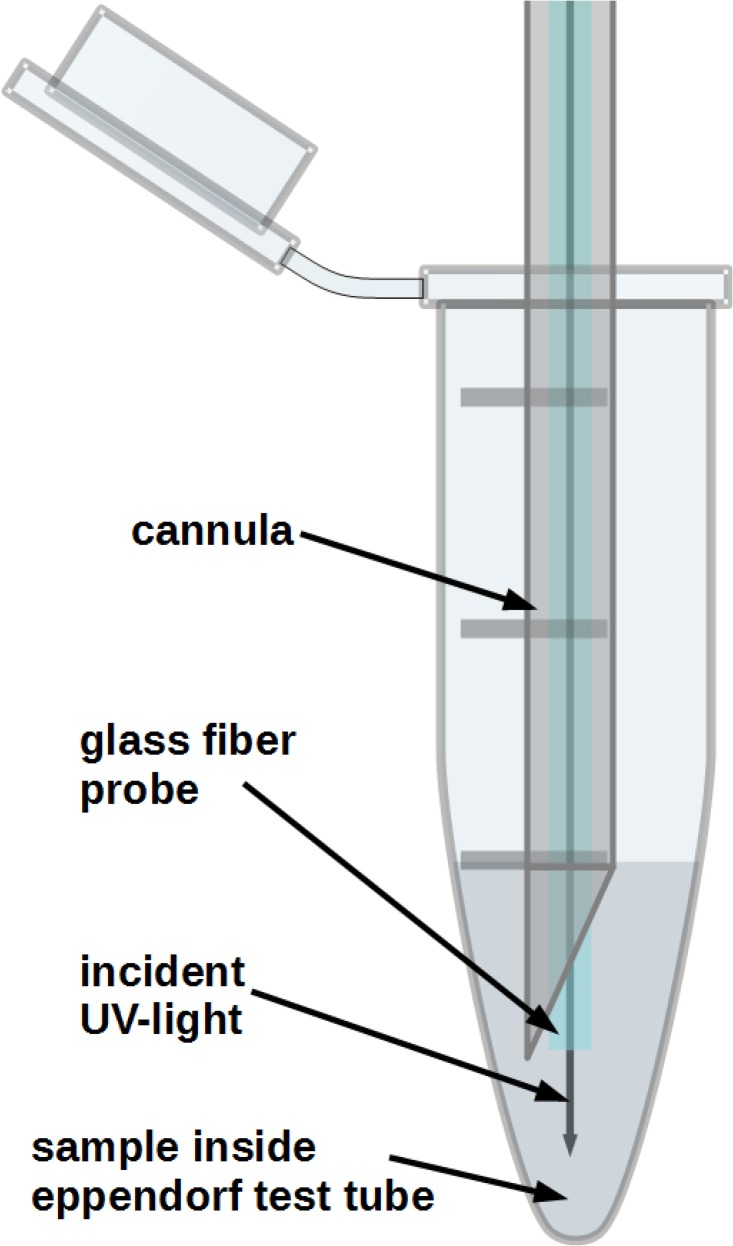
General setup for fluorescence measurements from the samples.

Following the measurements with the Cary the position of the tunable filter was set to 57° towards the incident fluorescence beam. At this angle the position of the edge of the longpass filter was located directly at 625 nm yielding high reflectance for light below 625 nm, which would be reflected in the direction of PD1 and a high transmittance for light with a wavelength above 625 nm which would reach PD2. In accordance with the position of the main emission peaks of PPIX and CPIII, in the set of samples with a changing CPIII concentration PD1 would yield a bigger change in signal intensity than PD2 with increasing CPIII concentration. In the experiments with changing PPIX concentration PD2 would detect a bigger change in intensity than PD1. This difference would allow us to qualitatively detect changes in porphyrin concentrations in living samples for following studies since the buildup of concentrations of CPIII and PPIX are necessarily also linearly correlated. For the sample measurements the tip of the glas fiber was threaded into an injection needle cannula, positioned central in the test tube and held in position with a conventional helping-hand as is used for soldering. The tip of the optical fiber was dipped 5 mm below the surface of the sample solutions leaving approximately 700 to 750 *μ*l of the sample volume to be fully irradiated and the fluorescence was measured via the back channel (Fig 5).

Following our previous work we learned that longer irradiation times at 405 nm, with the used irradiance of 30 *mW*/*cm*^2^ could cause significant decrease in fluorescence intensity, therefore that irradiation time was drastically reduced [14]. Each sample was measured for 30–40 seconds and the position of the needle was always the same. Each experiment consisted of 4 different samples, with differing concentrations and the experiments could be repeated with 3 different sets of these 4 samples. After all experiments had been performed with our device, tinfoil was used to shield the samples from ambient light and the samples were each measured in the Cary Eclipse Fluorescence Spectrophotometer again.

## Results

**Fig 6:**
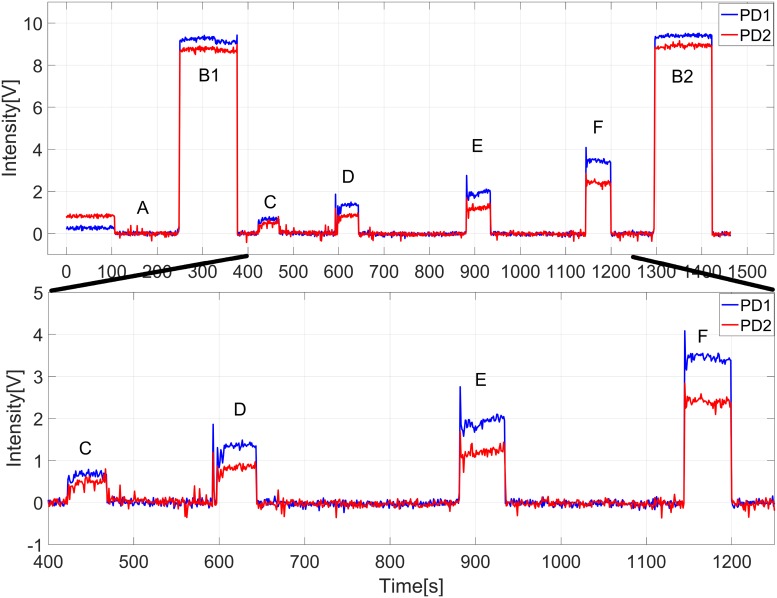
Raw data acquired with our filter-fluorometer: Exemplary for one measurement of a mixture with changing CPIII concentration. The measurement begins with an offset (A), PMMA sample (B1 and B2) and the 4 different mixtures (C–F).

Each measurement with our filter fluorometer was repeated 3 times with the same sample. The irradiation time and intensity showed no significant difference in fluorescence emission in the three repetitions of the measurements. Fig 6 shows one measurement of CPIII as the raw data and the segment finally used, with voltage as a function of time. It represents one single measurement of CPIII with the red line showing the signal received by PD2 (625–652 nm, the higher wavelength range) and the blue line representing the signal received by PD1 (607–625 nm, the lower wavelength range). The main purpose of the PMMA sample measurements (B1 and B2) was to confirm the measurement conditions and the setting of the device at the begin and the end of each test. A is the offset and C–F are the measured signals from 1 (lowest concentration) to 4 (highest concentration). What we can see is the intensity overall increases linearly from one sample to the next.

**Fig 7:**
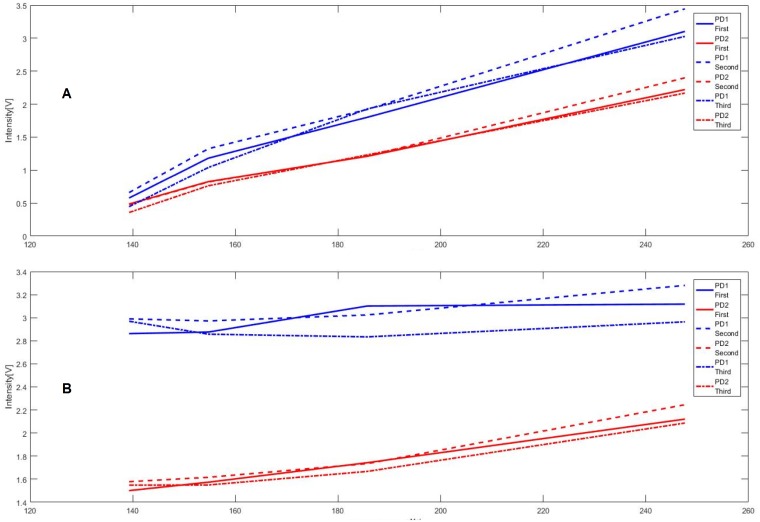
Changing CPIII concentration (A), Changing PPIX concentration (B). First measurement line, second measurements dotted line and third measurement fine dotted line.

Fig 7 shows the averages of the measurements seen as a function of the concentration in *μ*Mol/l for both types of samples separately (Fig 7A and 7B) and the development of the differences between the signal received for PD1 and PD2. Each of the single points represents the average of one set of data points recorded over 30–40 seconds at the given time. The measurements were conducted three times to compare the variation between each measurement (Fig 7A and 7B, PD1 First—Third and PD2 First—Third). What can already be seen in the averages with changing concentration is that the signals received in both PDs change with a different slope. When the CPIII concentration changes in a mixture with constant PPIX concentration, the signal in PD1 rises faster than the signal in PD2. Although the intensity of fluorescence increases due to the increase of porphyrins overall, the intensities of the emitted fluorescence in both wavelength ranges change at a different pace.

**Fig 8:**
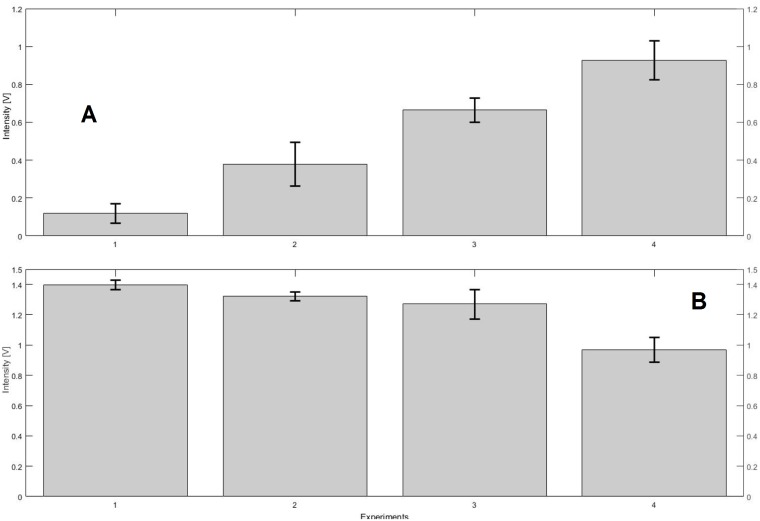
Differences changing CPIII concentration (A), differences changing PPIX concentration (B). **(CPIII A)** 1: 0.12V ± 0.052V, 2: 0.38V ± 0.11V, 3: 0.67V ± 0.063V, 4: 0.93V ± 0.102V, **(PPIX B)** 1: 1.4V ± 0.032V, 2: 1.32V ± 0.03V, 3: 1.27V ± 0.097V, 4: 0.97V ± 0.082V.

The higher importance lies with the behavior of both received signals to each other. When the signal received by PD2, relating more to a range where the peak of PPIX lies changes faster then this means a change in concentration of PPIX. In the other case, if there is a stronger change in PD1, which represents the signal stemming from the range where the peak of CPIII lies, then it naturally means a change in concentration of CPIII. This can be easily expressed with the change in difference between both PDs (Fig 8). The average differences between the signals received by both PDs change drastically from the lowest concentration to the highest.

**Fig 9:**
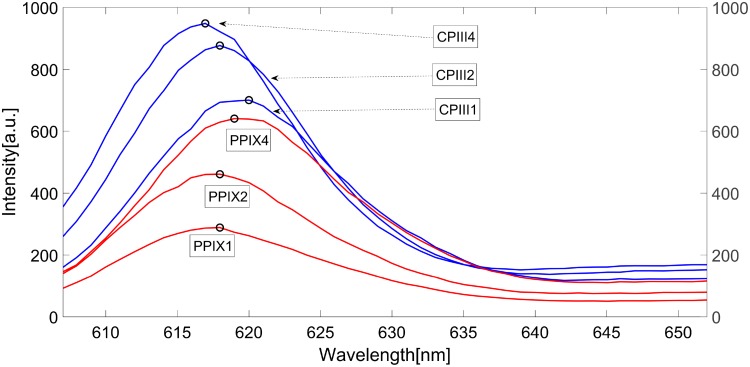
Control of the spectra of the samples as measured with the Cary spectro-fluorometer.

To confirm our measurements and our interpretation of the results, all samples were measured with the spectro-fluorometer again and plotted together (Fig 9). the samples containing a changing PPIX concentration show a slow decrease in difference when measured with our device. This behaviour indicates an increase of emission intensity in the upper wavelength range. When measured in the spectro-fluorometer the peak of the spectrum must hence also move towards the upper wavelength range with increasing PPIX concentration, which can be seen in Fig 9 (PPIX 1-4). On the other hand, the samples containing a changing CPIII concentration show a stronger increase of difference between PD1 and PD2. The control with the spectro-fluorometer here shows a movement of the peak towards the lower wavelength range Fig 9 (CPIII 1-4). With our device this movement appears as an increase of intensity difference between PD1 and PD2. It becomes obvious that the growing fraction of the mixture pushes the peak of the fluorescence emission into the direction of the peak of the pure porphyrin samples (see Fig 4). The measurements in the spectrofluorometer showed no shifts in the spectrum as would have been expect if significant aggregation of porphyrins had occurred.

Photobleaching would show as an overall decrease in fluorescence intensity as the porphyrins react with singlet oxygen and show no emission, lowered emission intensity or shifted bands due to formation of photoproducts. A significant decrease in intensity could not be observed in 3 consecutive measurements of the same samples. In our previous studies we observed this decrease in intensity over several measurements which led us to decrease the irradiation time to 30–40 seconds for each measurement. Furthermore photobleaching would likely show in the followup measurements with the spectrofluorometer within the spectral ranges measured which did not happen (Fig 9). We did not observe additional peaks or spreading of the peaks as would be expected in the presence of additional products of reactions with singlet oxygen.

## Discussion

Our device allows us to reliably detect increasing levels of porphyrins with peaks in the targeted range between 607 and 652 nm. In our previous studies we were already able to show the linear correlation between increasing porphyrin concentration and increased intensity of the fluorescence emission [14]. The division of the spectrum between the lower and upper wavelength range at 625 nm allows us to detect changes of the two targeted porphyrins, PPIX and CPIII even in mixtures. With our device these concentration changes appear as changes in difference between the signals recorded by PD1 and PD2. An increase in difference indicates an increasing CPIII concentration. A decrease in difference indicates an increase of PPIX concentration. Our device allows a qualitative measurement of changing compositions in porphyrin solutions and a relative quantitative measurement of total porphyrin concentration. The lowest changing concentration used was 15.47 *μ*Mol/l, which can also be seen as the current proven sensitivity of our fluorometer. Literature data for amounts of PPIX that can be expected in living cancer cells point towards 69 *μ*Mol/l [2]. The following steps need to focus on using samples of cancer cells to detect changes and test different approaches to treatment and measure the cell kill ratios using our device. Since the decreased activity of the enzyme Ferrochelatase has been reported for several types of cancer our future work will focus on comparisons between different cancer cell lines. As our device is also meant to bridge the limitations of conventional Photodynamic Therapy by approaching the target via an optical fiber, we will test the cancer cells as microtissues. So far we have only provided successful measurements under static conditions, with known concentrations of porphyrins. Dynamical changes need to be provided by changing concentrations within living cells as PPIX and its precursors are created in the heme synthesis, which can be shown by fluorescence measurements [15].

## Supporting information

S1 Data 11102017 57° first CPIII second PPIX three times. (TXT)

S2 Data 12102017 57° first CPIII changing. (TXT)

S3 Data 12102017 57° mixture 1-1. (TXT)

S4 Data 12102017 57° mixture 1-2 and 1-3. (TXT)

S5 Data 13102017 57° first CPIII changing b. (TXT)

S6 Data 13102017 57° first CPIII changing second measurement 40 seconds. (TXT)

S7 Data 13102017 57° first CPIII changing. (TXT)

S8 Data 13102017 57° first PPIX changing first. (TXT)

S9 Data 13102017 57° first PPIX changing second. (TXT)

S10 Data 13102017 57° first PPIX changing third. (TXT)

S11 Data FF02-632-22 0°. (TXT)

S12 Data FF02-632-22. (TXT)

S13 Data TLP01-704 57° reflection. (TXT)

S14 Data TLP01-704 57° transmission. (TXT)

S15 Data FF650-Di01. (TXT)

S16 Data 171011ppIXcpIII247mikroM. (XLS)

S17 Data 171025CPIII-1+2. (CSV)

S18 Data 171025CPIII-3+4. (CSV)

S19 Data 171025PPIX-1+2. (CSV)

S20 Data 171025PPIX-3+4. (CSV)

## References

[1] Rapozzi V, Jori G. Basic and Clinical Aspects of Photodynamic Therapy In: Resistance to Targeted Anti-Cancer Therapeutics. Springer International Publishing; 2014 p. 3–26. Available from: 10.1007/978-3-319-12730-9_1.

[2] Nakayama T, Otsuka S, Kobayashi T, Okajima H, Matsumoto K, Hagiya Y, et al Dormant cancer cells accumulate high protoporphyrin IX levels and are sensitive to 5-aminolevulinic acid-based photodynamic therapy. Scientific Reports. 2016;6(1). 10.1038/srep36478 PMC5114660 27857072

[3] Dysart JS, Patterson MS. Characterization of Photofrin photobleaching for singlet oxygen dose estimation during photodynamic therapy of MLL cells in vitro. Physics in Medicine and Biology. 2005;50(11):2597–2616. 10.1088/0031-9155/50/11/011 15901957

[4] Sailer R, Strauss WSL, Wagner M, Emmert H, Schneckenburger H. Relation between intracellular location and photodynamic efficacy of 5-aminolevulinic acid-induced protoporphyrin IXin vitro. Comparison between human glioblastoma cells and other cancer cell lines. Photochem Photobiol Sci. 2007;6(2):145–151. 10.1039/b611715e 17277837

[5] Inoue H, Kajimoto Y, Shibata MA, Miyoshi N, Ogawa N, Miyatake SI, et al Massive apoptotic cell death of human glioma cells via a mitochondrial pathway following 5-aminolevulinic acid-mediated photodynamic therapy. Journal of Neuro-Oncology. 2007;83(3):223–231. 10.1007/s11060-006-9325-8 17245620

[6] Morton C, Szeimies RM, Sidoroff A, Wennberg AM, Basset-Seguin N, Calzavara-Pinton P, et al European Dermatology Forum Guidelines on topical photodynamic therapy. European Journal of Dermatology. 2015;25(4):296–311. 10.1684/ejd.2015.2570 26065545

[7] Ji Z, Yang G, Vasovic V, Cunderlikova B, Suo Z, Nesland JM, et al Subcellular localization pattern of protoporphyrin IX is an important determinant for its photodynamic efficiency of human carcinoma and normal cell lines. Journal of Photochemistry and Photobiology B: Biology. 2006;84(3):213–220. 10.1016/j.jphotobiol.2006.03.006 16709459

[8] Pilný J, Kopečná J, Noda J, Sobotka R. Detection and Quantification of Heme and Chlorophyll Precursors Using a High Performance Liquid Chromatography (HPLC) System Equipped with Two Fluorescence Detectors. BIO-PROTOCOL. 2015;5(3).

[9] Cohen D, Lee P. Photodynamic Therapy for Non-Melanoma Skin Cancers. Cancers. 2016;8(10):90 10.3390/cancers8100090 PMC5082380 27782043

[10] Hillemanns P, Weingandt H, Baumgartner R, Diebold J, Xiang W, Stepp H. Photodetection of cervical intraepithelial neoplasia using 5-aminolevulinic acid-induced porphyrin fluorescence. Cancer. 2000;88(10):2275–2282. 10.1002/(SICI)1097-0142(20000515)88:10%3C2275::AID-CNCR11%3E3.0.CO;2-B 10820349

[11] Hindmarsh JT, Oliveras L, Greenway DC. Biochemical differentiation of the porphyrias. Clinical Biochemistry. 1999;32(8):609–619. 10.1016/S0009-9120(99)00067-3 10638943

[12] Huang W, Liu Q, Zhu EY, Shindi AAF, Li YQ. Rapid simultaneous determination of protoporphyrin IX, uroporphyrin III and coproporphyrin III in human whole blood by non-linear variable-angle synchronous fluorescence technique coupled with partial least squares. Talanta. 2010;82(4):1516–1520. 10.1016/j.talanta.2010.07.034 20801366

[13] Swietach P, Vaughan-Jones RD, Harris AL, Hulikova A. The chemistry, physiology and pathology of pH in cancer. Philosophical Transactions of the Royal Society B: Biological Sciences. 2014;369(1638):20130099–20130099. 10.1098/rstb.2013.0099 PMC3917353 24493747

[14] Landes R, Illanes A, van Oepen A, Goeppner D, Gollnick H, Friebe M. Fiber-optic filter fluorometer for emission detection of Protoporphyrin IX and its direct precursors—A preliminary study for improved Photodynamic Therapy applications. Results in Physics. 2018;8:1232–1233. 10.1016/j.rinp.2018.01.059

[15] Dysart JS, Patterson MS. Photobleaching kinetics, photoproduct formation, and dose estimation during ALA induced PpIX PDT of MLL cells under well oxygenated and hypoxic conditions. Photochem Photobiol Sci. 2006;5(1):73–81. 10.1039/b511807g 16395430

